# Properties of Uncommon Indirect Immunofluorescence Staining Patterns Determined during Antinuclear Antibody Detection on HEp-2 Cells

**DOI:** 10.3390/jcm10173866

**Published:** 2021-08-28

**Authors:** Nada Tomić Sremec, Ana Kozmar, Josip Sremec, Branimir Anić, Drago Batinić

**Affiliations:** 1 Department of Laboratory Diagnostics, University Hospital Centre Zagreb, 10000 Zagreb, Croatia; ana.kozmar@kbc-zagreb.hr (A.K.); drago.batinic@mef.hr (D.B.); 2 Department of Neurology, University Hospital Sveti Duh, 10000 Zagreb, Croatia; joza_sremec@yahoo.com; 3 Department of Internal Medicine, University Hospital Centre Zagreb, 10000 Zagreb, Croatia; branimir.anic@mef.hr; 4 School of Medicine, University of Zagreb, Šalata 3, 10000 Zagreb, Croatia

**Keywords:** antinuclear antibodies, indirect immunofluorescence, HEp-2, autoimmunity

## Abstract

In this study, we aimed to assess the prevalence of uncommon staining patterns found during testing for the presence of antinuclear antibodies (ANA) and to determine their association with certain antibodies and clinical diagnoses. Presence of ANA and the staining pattern was determined in 10955 samples using indirect immunofluorescence (IIF) on HEp-2 cells. ANA-positive samples were assessed for presence of 14 specific antibody types using a microbead based system. Demographic data (age, sex) and clinical diagnoses were collected from the referral documentation. Particular staining patterns were then compared with a representative comparison group comprised of samples with common staining patterns using these criteria. There were 22 patterns present in less than 3% of samples each and these were jointly present in 42.43% of ANA-positive samples. Specific antibodies were found in proportions similar to the comparison group (46.06%) and varied significantly between patterns. Likewise, there were significant differences in antibody distribution in particular patterns. Some patterns were associated with presence of rheumatic diseases or inflammatory arthropathies, while in others there was a concurrent diagnosis of liver disease, or a neoplastic process. Many of the uncommon IIF patterns have distinctive characteristics that warrant further investigation in order to determine their role in diagnosing various diseases, not limited only to the illnesses of the rheumatic spectrum. IIF on HEp-2 cells remains an irreplaceable method because of the diversity of ANA, only a number of which can be detected using other standardised methods.

## 1. Introduction

Ever since their discovery, antibodies directed against cellular antigens, commonly referred to as antinuclear antibodies (ANA), have been a significant tool in the diagnostic approach to patients with a suspicion of autoimmune illness [1,2]. Their assessment is one of the crucial steps in the evaluation and diagnostic workup of patients with systemic autoimmune rheumatic diseases (SARD) such as systemic lupus erythematosus (SLE) (where the presence of ANA at any point in the disease serves as an entry criterion for diagnosis) [3,4,5], Sjögren’s syndrome [6,7], systemic sclerosis [8], dermatomyositis/polimyositis [9] and others. Historically, those antibodies were thought of as effectors involved in the pathogenetic processes involved in the genesis of autoimmune rheumatic diseases [10], but later it has been hypothesised that they are in many cases an epiphenomenon of the disease, caused by elevated exposure of intracellular antigens to the immune system due to enhanced or aberrant apoptotic processes, or delayed elimination of those antigens from the system [11].

Despite of recent advancements in developing commercial assays based on a pre-defined mixture of cellular antigens [12,13,14], the preferred method for the initial assessment of ANA is still indirect immunofluorescence (IIF) on HEp-2 cells [15]. This method allows, provided that the competence of personnel performing the test is adequate, the detection of the presence of any autoantibody directed against cellular antigens, even of unknown significance or specificity, rather than a finite number of known common antigens [16,17]. When performing the test, a number of immunofluorescence patterns can be observed, of which some are associated with certain specific autoantibodies. One of the most thoroughly investigated examples are antibodies directed against double stranded DNA (anti-dsDNA antibodies), that are known to typically produce a homogeneous pattern on IIF performed on HEp-2 cells [18]. In some cases, the association between certain IIF patterns and specific autoantibodies is rather low, and additional tests are usually performed to determine the type of specific autoantibody. Those tests usually assess the presence of a number of common autoantibodies associated with autoimmune diseases [19,20]. The negative result of a confirmatory test does not exclude the existence of an unknown antibody type, and leaves both clinicians and laboratory personnel with a dilemma regarding the significance of a positive IIF test in these cases.

According to the currently valid international consensus, the International Consensus on ANA Patterns (ICAP) [21], there are 29 recognised discrete HEp-2 cell IIF patterns (Table 1).

Those patterns are divided into nuclear, cytoplasmic and mitotic subtypes, with a designated level of competence required for the recognition of each particular pattern [22]. There is a number of recognised IIF patterns that are seldom reported, partly due to their rarity, and possibly partly due to the lack of experience of the laboratory personnel performing the test [23,24]. These less common IIF patterns are in many cases of unknown significance. In some instances, evidence exists for their association with diseases outside the rheumatic spectrum, such as with the rods and rings pattern [25]. On the other hand, at least one pattern, known as the dense fine speckled pattern, associated with DFS70/lens epithelium-derived growth factor (LEDGF) specific antibodies, seems to exclude the presence of ANA-associated rheumatic diseases [26].

The aim of this study is to assess the prevalence of unusual IIF patterns on HEp-2 cells in a setting of a tertiary facility with a high throughput of patients referred for the detection of ANA, to assess main demographic characteristics of the patients in whom they are observed, and to comprehensively analyse their potential associations with particular specific autoantibodies and clinical diagnoses.

## 2. Materials and Methods

The samples in this study were taken from 10,955 consecutive patients referred to our institution for detection of ANA in a period of 12 months. Samples were collected according to regular procedures: venous blood (4 mL) was collected into standard clot activator serum vials, and serum was separated after a 30 min rest at room temperature by centrifuging at 3750 rpm for 10 min. Sera were stored at −20 °C until analysis. Data regarding patients’ sex and age, as well as the clinical diagnoses were acquired from the documentation made available upon their referral. The design of this study was approved by the institutional ethics committee (approval No 8.1-18/238-2, 02/21 AG), and is in concordance with the Helsinki Declaration.

The presence of ANA was determined in sera using indirect immunofluorescence assay on HEp-2 cells (EUROIMMUN Medizinische Labordiagnostika AG, Lübeck, Germany), in 1:100 dilution. This dilution was chosen as a cut-off yielding 5% positive results in the local healthy population, in accordance with international recommendations [15]. This methodology uses manual incubation of sera with slides coated with cell substrate. Antibodies in sera, if present, react with antigens present in those cells. After washing, slides are incubated with FITC (fluorescein isothiocyanate) conjugated antibodies that bind to patient’s antibodies and are subsequently visible under a fluorescent microscope (Olympus BX51, Olympus, Japan).

ANA-positive samples were then analysed for the presence of 14 specific autoantibodies (anti-dsDNA, anti-SS-A (60 kDa), anti-TRIM21, anti-SS-B, anti-Sm, anti-Sm/RNP, anti-Scl-70, anti-Jo-1, anti-ribosomal proteins, anti-centromere (CENP-B), anti-PmScl, anti-PCNA, anti-histone, and anti-U1-RNP) using the FIDIS™ Connective Profile microbead based system (Theradiag, Croissy Beaubourg, France). This system utilizes polystyrene microbeads coated with antigens that bind to specific antibodies if they are present in a patient’s serum. After this initial incubation, the antibody-bead complex is incubated with PE-conjugated anti-Fc antibodies. The resultant complex is then available for analysis via Luminex^®^ 100/200™/xMAP^®^ technology (Luminex Corporation, Austin, TX, USA), in which two laser beams (one for qualitative and other for quantitative analysis) are used to assess the sample.

Pattern type was determined in each sample according to the criteria and guidelines postulated within the ICAP consensus [22]. In patients with multiple concurrently present IIF patterns, the most prominently visible pattern was designated as the primary pattern, while the number and type of other patterns present in the sample was noted as well. All IIF patterns were determined and verified by two experts with considerable experience in the field, being the only personnel responsible for performing ANA IIF analyses in a tertiary facility (national referral centre) with a yearly throughput of 10,000–20,000 samples referred for ANA detection. Our facility is accredited for these diagnostic procedures by the national accreditation agency, and complies with the ISO 15,189:2012 standard. Our procedures and results are frequently validated through international external quality control mechanisms, such as UK NEQAS.

Samples with patterns occurring in less than 3% of total 10,955 samples were then included in further analyses. This cut-off was chosen because of the much higher prevalence of the remaining IIF patterns. Prevalence of particular rare patterns was assessed, as well as their association with patients’ sex and age. The association of particular patterns with specific autoantibodies and their prevalence in patients with specific clinical diagnoses were assessed as well.

Clinical diagnoses were divided into four subcategories according to the ICD (international classification of diseases)-10 [27] codes assigned to the patients in their referral documentation; wherein inflammatory arthropathies comprised diagnoses ranging from M05 to M14, systemic connective tissue disorders had codes from M30 to M36, neoplasms from C00 to D48 and liver diseases from K70 to K77. The remainder of cases had various sporadic diagnoses. Patients were referred either by an immunology or rheumatology specialist, or by a general practitioner (often, but not exclusively after recommendation issued by aforementioned specialists).

Samples with particular IIF patterns were additionally compared to a representative comparison group comprised of 219 consecutive ANA-positive samples from which aforementioned rare IIF patterns were excluded. This comparison group comprised samples with commonly found IIF patterns. Among those samples, the most commonly found pattern was AC-1, with 52.51%, followed by AC-4, found in 27.85% of samples. The remaining patterns were distributed evenly between AC-3, AC-5, AC-8, AC-20 and AC-21 patterns. Samples in this group underwent the same procedures as the study group: after determining the IIF pattern, antibody specificity was determined using the aforementioned FIDIS™ connective profile system, and clinical as well as demographic characteristics were obtained.

The statistical analysis was performed using Statistica software (version 13.3.0., TIBCO Software Inc., Palo Alto, CA, USA). Depending on the distribution (determined using the Kolmogorov–Smirnov test for normality) and variable type, data were presented as mean ± SD or median ± IQR and analysed using appropriate tests. Statistical significance was set to *p* < 0.05.

## 3. Results

Of 10,955 analysed samples, 4107 were ANA-positive, which represents 37.48% of all samples. Of those ANA-positive samples, rare patterns (conforming to the cut-off point of maximum presence in 3% of samples) were detected in 1743 cases (42.43% of ANA-positive samples). In 873 of those cases, there were other IIF patterns present simultaneously, while in the other 870 cases, rare patterns were solitary.

**Figure 1:**
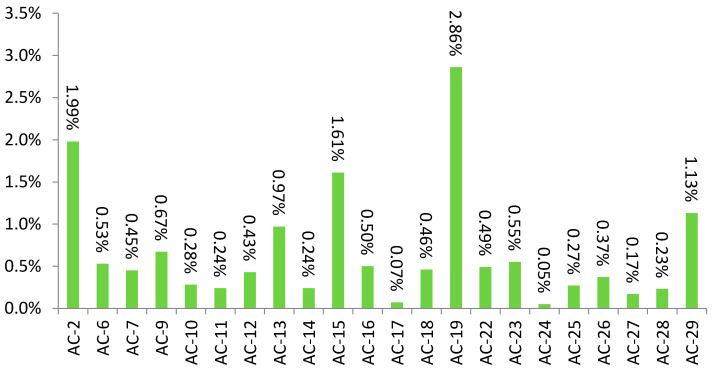
Percentages of ANA patterns with total occurrence frequency (in all tested samples) of less than 3%, determined using indirect immunofluorescence assay on HEp-2 cells in 1:100 dilution.

A total of 22 patterns complied with the rarity criterion (i.e., present in less than 3% of all tested samples) (Figure 1).

### 3.1. Demographic Characteristics

Compared with the comparison sample, in which age median was 56 [IQR 43–67] years, age medians differed statistically in patients with AC-2 (52 [IQR 37–62] years), AC-7 (43 [IQR 31–57.25] years), AC-10 (48 [IQR 33.25–59] years) and AC-13 (50 [IQR 38–62] years), *p* < 0.0001). Sex distribution, compared with the comparison group in which there were 181 female and 38 male patients, differed for patients with AC-9 (43 female and 30 male patients, *p* < 0.0001), AC-15 (124 female and 53 male patients, *p* = 0.0038), AC-16 (31 female and 24 male patients, *p* = 0.0001), AC-19 (232 female and 82 male patients, *p* = 0.0202), AC-22 (35 female and 19 male patients, *p* = 0.008) and AC-24 patterns (4 male and 2 female patients, *p* = 0.012).

### 3.2. Specific Autoantibodies

**Figure 2:**
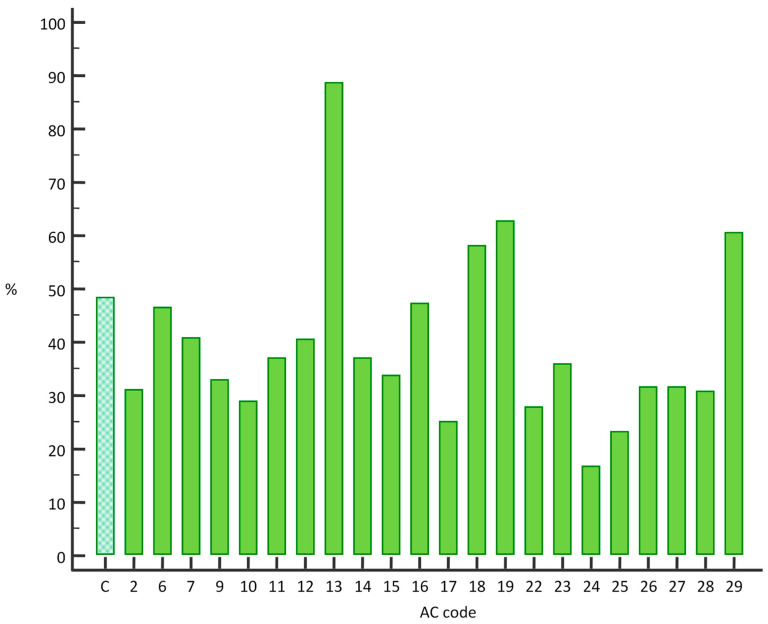
Percentage of samples with detected specific autoantibodies using Theradiag FIDIS™ connective profile in patients with particular rare IIF patterns determined using indirect immunofluorescence assay on HEp-2 cells in 1:100 dilution. C = comparison group.

In 803 samples with rare IIF patterns we detected the presence of one or more specific autoantibodies (46.06%), a percentage that is quite similar to proportions found in the comparison group (48.40%). Specific autoantibodies were detected less frequently in samples with AC-2 (68/211 cases, *p* = 0.0003), AC-9 (24/73 cases, *p* = 0.0215), AC-15 (60/177 cases, *p* = 0.0041), AC-22 (15/54, *p* = 0.089), and AC-25 (7/30, *p* = 0.0108) patterns, while in samples with AC-13 (93/105 cases, *p* < 0.0001), AC-19 (197/314, *p* = 0.0013) and AC-29 (86/142, *p* = 0.0307) patterns, those autoantibodies were detected more frequently (Figure 2).

In the comparison group, anti-dsDNA antibodies were found in 14.61% of samples, anti-SS-A (60 kDa) antibodies were found in 10.50%, anti-TRIM21 in 14.15%, anti-SS-B in 7.31%, anti-Sm in 0.46%, anti-Sm/RNP in 2.74%, anti-Scl-70 in 2.74%, anti-Jo-1 in 0.91%, anti-ribosomal proteins in 0.91%, anti-centromere in 5.48%, anti-PmScl in 5.94%, anti-PCNA in 7.76%, anti-histone in 5.02% and U1-RNP in 7.31% of samples. In many particularly rare patterns, the distribution of one or more specific antibodies differed from the comparison group, as particular antibodies existed less or more frequently, as illustrated in Table 2.

### 3.3. Clinical Diagnoses

Clinical diagnoses that refer to SARD were present in 38 out of 219 patients in the comparison group (17.35%). Occurrence of these diagnoses was not significantly less common in patients with AC-2 codes (10.55%, *p* = 0.053). They were significantly more prevalent in patients with AC-13 IIF pattern (41/106, 38.68%, *p* < 0.0001), and less dramatically in patients with the AC-18 pattern (34%, *p* = 0.012). They were less common in patients with AC-16 (5.45%, *p* = 0.032), AC-22 (5.55%, *p* = 0.032), AC-23 (6.56%, *p* = 0.042), and AC-27 IIF patterns (0%, *p* = 0.049). There were no differences for any other IIF patterns according to this criterion.

Diagnoses of inflammatory arthropathies were present in 41 out of 219 patients in the comparison group (18.72%). None of the rare IIF patterns had significantly different proportions of these referring diagnoses.

As for patients with diagnoses of neoplastic processes, who accounted for 3 of 219 comparison group patients (1.37%), they were more common in patients with AC-12 IIF pattern (4/47, 8.51%, *p* = 0.020), as well as in AC-16 (12.72%, *p* = 0.0007) and AC-23 (6.56%, *p* = 0.043) patterns. These patients were uncommon across all of the analysed IIF patterns.

Liver diseases were the main referring diagnosis for 5/219 patients in the comparison group (2.28%). They were much more common in patients with the AC-6 pattern (13/58, 22.41%, *p* < 0.0001), as well as in patients with the AC-11 (4/27, 14.81%, *p* = 0.010) pattern, AC-12 pattern (10/47, 21.27%, *p* < 0.0001), AC-15 pattern (18/177, 10.17%, *p* = 0.001), AC-16 (3/8, 37.5%, *p* = 0.002) and AC-22 pattern (5/54, 9.26%, *p* = 0.028).

**Figure 3:**
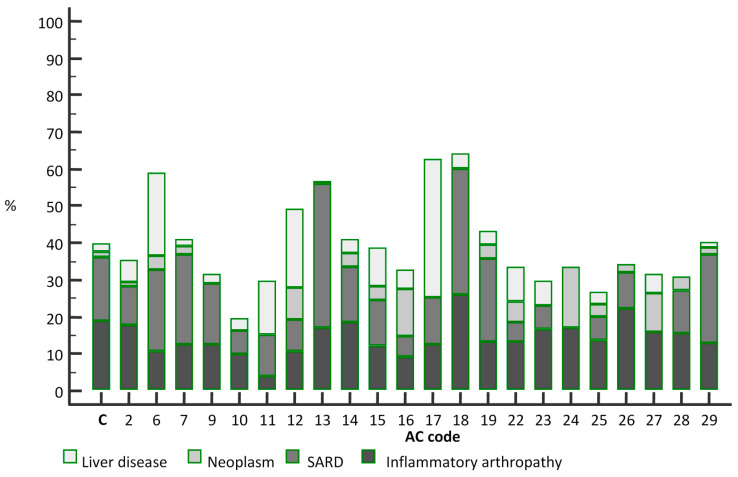
Percentage distribution of groups of diagnoses acquired from referral documentation in patients with particular rare IIF patterns determined using indirect immunofluorescence assay on HEp-2 cells in 1:100 dilution; C = comparison group.

Proportions of referral diagnoses in patients with each rare IIF pattern are given in Figure 3.

In order to illustrate the specificities of the nuclear dense fine speckled pattern (AC-2), we performed a comparison with the cytoplasmatic dense fine speckled pattern, AC-19. Frequency of antibody detection varies greatly between these two groups, as antibodies were detected in 197/314 samples with the latter pattern (62.74%), compared with mere 68/218 samples in the AC-2 group (31.19%, *p* < 0.0001). Samples with the AC-19 pattern had a significantly higher occurrence of a plethora of antibodies, namely anti-dsDNA, anti-SS-A 60 kDa, anti-TRIM21, anti-histones (*p* < 0.0001 for all), anti-Jo-1 (*p* = 0.008), anti-ribosomes (*p* = 0.001), anti-Sm/RNP (*p* = 0.045), anti-PCNA (*p =* 0.002), anti-PmScl (*p* = 0.012) and anti-U1-RNP (*p =* 0.018). The most dramatic clinical differences between those groups were in relation to SARD, as diagnoses from this group were present in 22.61% of patients with the AC-19 pattern, compared with only 11.47% of patients in the AC-2 group (*p* = 0.0003). Other diagnoses were present in similar proportions.

In addition to all the rare IIF patterns recognised by the current nomenclature, in 124 samples we found fluorescence patterns that do not conform to any of the criteria in that nomenclature, nor do they represent a combination of any known IIF patterns. In 52 samples within this heterogenous group (41.93%), specific autoantibodies were found. Additional, simultaneously present common IIF patterns may account for a fraction of these specific autoantibodies; however, even when those novel patterns were solitary, specific autoantibodies were found in similar proportions (14/24, 58.33%).

### 3.4. Simultaneous Presence of Common Immunofluorescence Patterns

**Figure 4:**
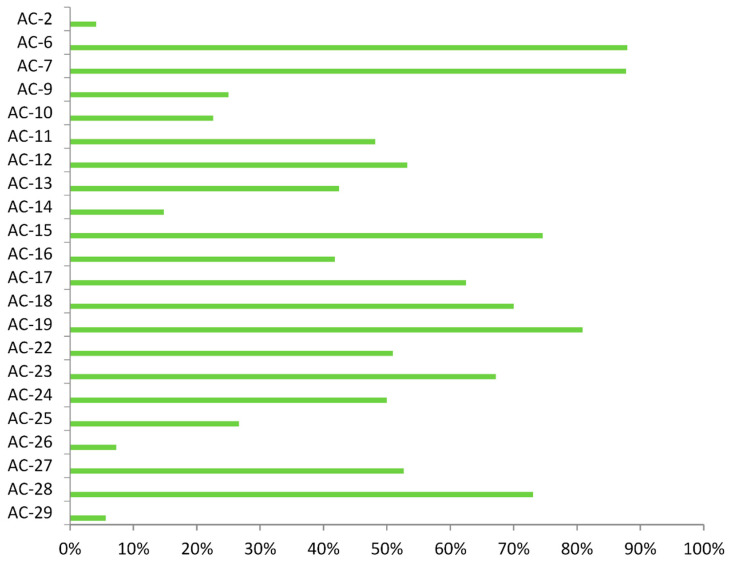
Percentage of samples of each rare IIF pattern with simultaneous presence of any common IIF pattern, determined using indirect immunofluorescence assay on HEp-2 cells in 1:100 dilution.

Proportions of samples with rare IIF patterns in which there was simultaneous presence of common IIF patterns varied greatly across the rare pattern range, as shown in Figure 4.

In order to determine if those common patterns were responsible for the presence of specific antibodies, we compared antibody positivity rates within each rare pattern, depending on presence of common patterns. In case of most patterns there were no differences between those groups, the exceptions being AC-9, AC-11, and AC-26, where there were more samples with specific antibodies when common patterns were present as well (61.11% vs. 26%, *p* = 0.011; 61.54% vs. 18.18%, *p* = 0.047; and 100% vs. 35.71%, *p* = 0.027, respectively).

## 4. Discussion

Recent standardisation efforts have without a doubt improved our understanding of the IIF method of ANA detection and the resulting staining patterns. It has also enabled researchers to assess the staining patterns that have until now been reported sporadically, without systematic research into their properties and significance.

In our study we utilised large numbers of samples referred to our facility for ANA testing to try to improve the knowledge of certain rare IIF patterns that are seen sporadically in everyday practice.

The proportions of rare patterns we have found demonstrate that even amongst uncommon patterns there are those that are found many times less frequently than the others. This prevented an objective assessment of their properties. For some patterns, we managed to show certain characteristics that differentiate them from other patterns.

The median age of patients both in the comparison group and in most of the study group, which is well above the age at which we might expect occurrence of systemic autoimmune diseases [28], might be explained by the tendency to refer patients to ANA diagnostics even in the absence of clear symptoms of autoimmune diseases, but rather in cases of otherwise unexplained symptoms and uncertain diagnoses. On the other hand, the immune system might exhibit greater autoimmune tendencies with older age, which might account for some of the positive ANA samples in our study [29]. Patients with several IIF patterns (AC-2, AC-7, AC-10, and AC-13) tend to be younger. For patients with the AC-2 pattern, this could be explained by its aforementioned disease-excluding properties, meaning that in our sample there could be a proportion of younger, healthy patients that have been referred to this type of diagnostic evaluation during routine or cautionary workups [26,30]. For AC-7 and AC-10 patterns, the reason for the younger patients’ age is less clear, owing to the comparative lack of their definite correlation to clinical entities, although our study demonstrated a tendency for anti-U1-RNP positivity in the AC-7-positive samples. This antibody is thought to be present in all cases of mixed connective tissue disorder (MCTD), as well as in some cases of SLE [31].

As for AC-13 pattern, the marginally younger age of these patients may be explained by the association of this staining pattern with SLE, although the validity of this association is still debated [32]. In our study, samples with this pattern were positive for specific autoantibodies in much greater proportion than the comparison group, and those antibodies were predominantly ones traditionally associated with Sjögren’s syndrome: anti-SS-A 60 kDa, anti-TRIM 21 (formerly known as SS-A 52 kDa) and anti-SS-B [33]. Recent studies suggest that antibodies that result in this staining pattern, anti-PCNA (proliferating cell nuclear antigen) antibodies, are associated with a variety of other conditions [34]. Of note is a significantly larger proportion of patients with this staining pattern who have referral diagnoses from the SARD category. However, results for this staining pattern have to be interpreted cautiously, as some combinations of common patterns can produce a similar IIF appearance.

AC-19 pattern, often found in the anti-synthetase syndrome [35], was the most common IIF pattern still conforming to our arbitrary 3% inclusion rule. In these samples, specific autoantibodies were more frequent than in the comparison group. Antibodies that accounted mostly for these positivity rates were anti-dsDNA, anti-TRIM21, anti-histones and anti-ribosomes. These antibodies have a common association with SLE [4,36,37].

We cannot adequately account for the presence of specific antibodies in patients with the AC-23 pattern, which was the case even when this easily distinguishable pattern was solitary. Perhaps these findings can be ascribed to the imperfect specificity of the used multiplex assay.

It is not surprising that amongst the samples with AC-29 pattern, fulfilling all the criteria from the international consensus [38], we found a fraction of samples positive for anti-Scl70 antibodies, specific for systemic sclerosis [39]. However, a significant proportion of those patients were positive for SLE specific [4] anti-dsDNA antibodies, with a homogenous IIF pattern being simultaneously present in only few of those samples.

Finally, we could not confirm that the AC-2 pattern was less commonly positive for specific autoantibodies, apart from anti-CEMP-B and anti-PCNA. It is possible that anti-DFS70/LEDGF antibodies would be positive in only a fraction of samples with this staining pattern, and that only the presence of this type of antibody suggests the absence of SARD, as proposed by earlier studies [29,40]. Samples without presence of these antibodies may instead be termed the “pseudo-DFS” pattern, as proposed by some authors [41].

Patients with this IIF pattern had a tendency for being less commonly diagnosed with SARD compared with the comparison group; however, statistical significance was not reached despite a sizeable number of such individuals (and evident only in comparison with patients with cytoplasmatic pattern of similar characteristics, AC-19), while for some patterns (AC-16, AC-22, AC-23, AC-27) those diagnoses were evidently less common despite the smaller sample size. On the other hand, there was a significant number of referrals for SARD in patients with the AC-18 IIF pattern, a pattern that has not been systematically investigated [42], and that has yielded a significant proportion of samples positive for anti-U1-RNP antibodies in our study, ubiquitous in MCTD [31]. 

AC-12 pattern was not only more commonly associated with liver diseases, which is to be expected given its known association with primary biliary cholangiitis [43], but with neoplastic conditions as well.

Other patterns associated with liver diseases were AC-6, AC-11, AC-15, AC-16 and AC-22. Many of the antibodies associated with these patterns were already implied in this setting [44,45,46,47,48].

Neoplastic disorders were more commonly present in patients with AC-16 and AC-23 codes. For the former, this is, per our knowledge, unreported to date. As for patients with the AC-23 pattern, it would be reasonable to hypothesise that this pattern could be iatrogenic, induced during treatment for neoplastic disorders, as it is thought to be a model of drug-induced antibody generation (most commonly ribavirin/interferon –α) [25,49].

Some of our results need to be interpreted in the context of simultaneously present common IIF patterns; however, it is likely that this is not an overwhelmingly significant factor, as we showed with our comparison of specific antibody presence depending on the presence of common IIF patterns.

The limitations of this study include the fact that some of the IIF patterns are extremely uncommon, and scarcely present even in a large sample such as in our study. That, combined with the fact that some of the specific autoantibodies determined by the method used in our study are rare as well, impairs the possibility to determine some of the possible pattern–antibody associations. The subjectivity of the IIF method may result in some inaccuracies as well [23], given that some common patterns can in lower titers be misinterpreted as rare patterns (e.g., AC-13). The type and manufacturer of IIF slides can also possibly account for some differences in rare pattern phenotypes and prominence. The specificity of the multiplex assay for detection of specific antibodies has to be taken with a degree of cautiousness, due to the seemingly large amount of unexpected antibody positivity across the rare pattern range. The representativeness of the study group may be reduced by the fact that it is not a population sample, but rather a sample of patients referred to ANA diagnostic procedures for a reason, which is reflected in positivity rates that are higher than we would expect in the healthy population [50]. Our analyses of referral diagnoses may be confounded by the fact that referral diagnoses are not always made by a specialist in the relevant disease spectrum.

## 5. Conclusions

This study demonstrates why it is of paramount importance to continue screening for ANA using the proven manual method that is IIF microscopy on HEp-2 cells, a procedure that gains relevance only through adequate expertise and adherence to the latest recommendations. It is obvious that we cannot firmly link any IIF pattern to one specific autoantibody, and that the unpredictable nature of staining, stemming from the presence of ANA in a sample, results in a plethora of diverse patterns. Thus, it would be unadvisable to recommend omitting testing for specific autoantibodies even in the absence of common IIF patterns, as even the most unusual patterns have a similar proportion of specific autoantibody positivity. On the other hand, IIF tests are necessary for as long as we do not develop diagnostic procedures that would truly be able to assess the presence of every possible antibody directed against intracellular antigens, which seems unlikely for the foreseeable future. Further studies are warranted to elucidate the processes that in certain circumstances result in differing IIF patterns being produced by the same antibody. Conversely, a large proportion of ANA-positive samples are still unexplained, as many antibodies that produce staining patterns are often not detected by the pre-defined mixtures of antigens that are included in various multiplex systems widely used today.

Hence, it is essential to report the IIF pattern detected by fluorescent microscopy and proceed with assessing the presence of specific autoantibodies. Presently, only this combination of procedures provides us with sufficient information to assess this type of antibodies. Whether the result of a particular positive ANA test represents true pathology, or rather the result of mildly unbalanced normal metabolic processes, is necessarily deduced clinically. ANA testing, for now, represents a valuable asset in diagnosing various immune-related diseases, but only if done thoroughly and interpreted cautiously.

## Figures and Tables

**Table 1:** Nomenclature and classification of antinuclear antibody (ANA) indirect immunofluorescence (IIF) patterns on HEp-2 cells.

Category	Subdivision	2nd Subdivision	AC Designation
Negative			AC-0
			
Nuclear	Homogeneous		AC-1
	Speckled	Dense Fine Speckled	AC-2
		Fine Speckled	AC-4
		Large/Coarse Speckled	AC-5
		Topo I	AC-29
	Centromere		AC-3
	Discrete Nuclear Dots	Multiple	AC-6
		Few	AC-7
	Nucleolar	Homogenous	AC-8
		Clumpy	AC-9
		Punctate	AC-10
	Nuclear Envelope	Smooth	AC-11
		Punctuate	AC-12
	Pleomorphic	PCNA	AC-13
		CENP-F	AC-14
			
Cytoplasmic	Fibrillar	Linear	AC-15
		Filamentous	AC-16
		Segmental	AC-17
	Speckled	Discrete Dots	AC-18
		Dense Fine Speckled	AC-19
		Fine Speckled	AC-20
	AMA		AC-21
	Golgi		AC-22
	Rods and Rings		AC-23
			
Mitotic	Centrosome		AC-24
	Spindle Fibers		AC-25
		NuMA	AC-26
	Intercellular Bridge		AC-27
	Mitotic Chromosomal		AC-28

Modified from [21]. AC-29 was recognized as an additional nuclear speckled pattern after the first version of the nomenclature, resulting in its specific position in the subsequent versions.

**Table 2:** Differences in distribution of specific antibodies in samples with particular rare IIF patterns.

	N	dsDNA	SS-A 60	TRIM21	SS-B	Sm	Sm/RNP	Scl-70	Jo-1	Ribosomal	CENP-B	PmScl	PCNA	Histone	U1-RNP
AC-2	218										3 1.4%		5 2.7%		
AC-6	58														
AC-7	49				0%										
AC-9	73										0%				
AC-10	31	0%													
AC-11	27			0%											
AC-12	47														
AC-13	106		82 77.4%	75 67.9%	42 39.6%										
AC-14	27														
AC-15	177										2 1.1%				
AC-16	55														
AC-17	8														
AC-18	50														9 18%
AC-19	314	88 28.0%	63 20.1%	101 32.2%						21 6.7%				37 11.8%	
AC-22	54														
AC-23	61			2 3.3%											
AC-24	6														
AC-25	30														
AC-26	41														
AC-27	19														
AC-28	26														
AC-29	142	46 32.4%						23 16.2%			0%				

Amount and percentages of samples with particular specific antibodies determined using Theradiag FIDIS™ connective profile in patients with particular rare IIF patterns determined using indirect immunofluorescence assay on HEp-2 cells in 1:100 dilution, that are significantly different (*p* < 0.05) to their distribution in an unfiltered comparison sample acquired using the same methodology

## Data Availability

Data used in this study is available at https://figshare.com/articles/dataset/ananada_novo_mc1/14397590 (accessed on 20 June 2021).
